# Quality improvement in practice: improving diabetes care and patient outcomes in Aboriginal Community Controlled Health Services

**DOI:** 10.1186/1472-6963-14-481

**Published:** 2014-10-07

**Authors:** Alice Stoneman, David Atkinson, Maureen Davey, Julia V Marley

**Affiliations:** Launceston Clinical School, University of Tasmania, Locked Bag 1377, Launceston, TAS 7250 Australia; The Rural Clinical School of Western Australia, The University of Western Australia, 12 Napier Terrace, PO Box 1377, Broome, WA 6725 Australia; Kimberley Aboriginal Medical Services Council, 12 Napier Terrace, PO Box 1377, Broome, WA 6725 Australia; School of Medicine, University of Tasmania, 17 Liverpool Street, Hobart, TAS 7000 Australia

**Keywords:** Indigenous, Aboriginal, Torres Strait Islander, Diabetes, Quality improvement cycles, Primary health care

## Abstract

**Background:**

Management of chronic disease, including diabetes, is a central focus of most Aboriginal Community Controlled Health Services (ACCHSs) in Australia. We have previously demonstrated that diabetes monitoring and outcomes can be improved and maintained over a 10-year period at Derby Aboriginal Health Service (DAHS). While continuous quality improvement (CQI) has been shown to improve service delivery rates and clinical outcome measures, the process of interpreting audit results and developing strategies for improvement is less well described. This paper describes the evaluation of care of patients with type 2 diabetes mellitus (T2DM) and features of effective CQI in ACCHSs in the remote Kimberley region of north Western Australia.

**Methods:**

Retrospective audit of records for Aboriginal and Torres Strait Islander primary care patients aged ≥15 years with a confirmed diagnosis of T2DM at four Kimberley ACCHSs from 1 July 2011 to 30 June 2012. Interviews with health service staff and focus group discussions with patients post audit. Main outcome measures: diabetes care related activities, clinical outcome measures and factors influencing good diabetes related care and effective CQI.

**Results:**

A total of 348 patients from the four ACCHSs were included in the study. Clinical care activities were generally high across three of the four health services (at least 71% of patients had cholesterol recorded, 89% blood pressure, 84% HbA_1c_). Patients from DAHS had lower median cholesterol levels (4.4 mmol/L) and the highest proportion of patients meeting clinical targets for HbA_1c_ (31% v 16% ACCHS-3; *P* = 0.02). Features that facilitated good care included clearly defined staff roles for diabetes management, support and involvement of Aboriginal Health Workers, efficient recall systems, and well-coordinated allied health services. Effective CQI features included seamless and timely data collection, local ownership of the process, openness to admitting deficiencies and willingness to embrace change.

**Conclusions:**

Well-designed health care delivery and CQI systems, with a strong sense of ownership over diabetes management led to increased service delivery rates and improved clinical outcome measures in ACCHSs. Locally run CQI processes may be more responsive to individual health services and more sustainable than externally driven systems.

## Background

Management of chronic disease, including diabetes, is a central focus of most primary health care services in Australia and of Aboriginal Community Controlled Health Services (ACCHSs) in particular [1–3]. The aim of diabetes care is to reduce the risk of developing microvascular and macrovascular complications by reducing hyperglycaemia, hypertension, hypercholesterolaemia, obesity, physical inactivity and smoking [4]. Testing at regular intervals, combined with appropriate therapeutic and lifestyle changes, can reduce morbidity and mortality [5–19]. The self-management support approach where patients are actively engaged in health care decisions and healthy behaviours is integral to chronic disease management [20, 21]. In the Australian Aboriginal setting, self-published reports have shown that self-management support is effective if led by Aboriginal Health Workers (AHWs) and delivered in a culturally appropriate manner [22, 23]. This is supported by a larger body of literature worldwide which demonstrates that self-management support delivered by ‘community health workers’ in underserved populations of ethnic minorities can improve clinical outcomes in patients with diabetes [24, 25].

Engagement in cycles of continuous quality improvement (CQI) can assist health services to identify areas of deficiency and implement appropriate changes to improve chronic disease management. CQI in the Aboriginal health care setting has been shown to improve service delivery rates and clinical outcome measures in Aboriginal patients with diabetes [26–31] however the process of interpreting audit results and developing strategies for improvement is less well described.

This study follows on from long term CQI in diabetes management which demonstrated significant improvements in quality indicators over 10 years at the Derby Aboriginal Health Service (DAHS) in the remote Kimberley region of northern Western Australia (“the DAHS diabetes study”) [28]. The aim of this study was to compare service delivery and outcome measures between DAHS and other ACCHSs in the region and to identify strategies for improving diabetes care and the CQI process.

## Methods

DAHS and three of the six other Kimberley ACCHSs providing clinical services were approached by the Kimberley Aboriginal Medical Services Council (KAMSC) and agreed to participate in the study. KAMSC provides regional support for Kimberley ACCHSs including supporting the development and implementation of the evidence-based Kimberley Chronic Disease Therapeutic Protocols [32] as well as CQI [28]. A mixed methods approach combining quantitative and qualitative data was utilised.

The audit period was from 1 July 2011 to 30 June 2012. The audit included Aboriginal and Torres Strait Islander patients seen at the four ACCHSs who had a confirmed diagnosis of type 2 diabetes, were ≥15 years old, were regular patients of one of the participating ACCHSs, and were not on renal replacement therapy. Patients were considered ‘regular’ if 1) their electronic records showed: ≥2 visits recorded in a 24 month period preceding the date of the search; the participating ACCHS was listed as their ‘primary care provider’; and their address was listed as living in the community of the participating ACCHS or 2) the Aboriginal ACCHS staff identified them as ‘regular’ (to include people who do not access other services but may attend < 2 per two years).

MMEx (The University of Western Australia’s Centre for Software Practice, Perth, Australia), a patient information and recall system was introduced between February 2010 and March 2011 across the four ACCHSs. MMEx incorporates patient progress notes, observation charts, pathology results, medications, care plans, an auditing tool, and a secure messaging service. Data for the audit was extracted from MMEx and transferred into Excel 2007 (Microsoft, Seattle, USA). Outliers were identified and data entry errors where they had occurred were corrected. The data were then imported into Stata, version 12 (StataCorp, Texas, USA).

Measures expected to be checked at least 6 monthly (blood pressure [BP], waist circumference, weight, glycated haemoglobin [HbA_1c_], brief foot checks and assessment of diet and physical activity) were assessed from 1 January 2012 to 30 June 2012. Measures expected to be recorded annually (urine albumin–creatinine ratio [ACR], estimated glomerular filtration rate [eGFR], total cholesterol, liver function tests [LFT], urine protein, retinal screening, full foot checks, smoking status assessment and general practice management plans [GPMP]) were assessed over the full audit year.

### Clinical outcomes measures

Overall median HbA_1c_, BP, cholesterol and ACR levels were determined using medians for individual patients. Proportions of patients with median HbA_1c_ and cholesterol levels meeting recommend targets were then determined [33]. As the recommended target for BP includes meeting the target for both systolic (≤130 mmHg) and diastolic (≤80 mmHg) measurement, the proportions of patients with at least half their electronic measurements meeting both targets were determined.

### Statistical analysis

DAHS was used as a benchmark in this study due to the previously published 10 year history of CQI of diabetes care [28]. Results from ACCHS-2 to −4 were compared with DAHS using the *χ*^2^ test for service activity, and the Mann–Whitney test for clinical outcome measures. Analysis was performed using Stata, version 12. *P* < 0.05 was considered statistically significant.

### Interviews and focus groups

Health care staff from the four ACCHSs were invited to participate in interviews after attending a presentation of the initial audit results. Interviews were semi-structured and covered topics including validity of the audit results, the service delivery system design, self-management support and community engagement. A total of 19 interviews were conducted involving nine AHWs, seven registered nurses (RNs), and three general practitioners (GPs). Most interviews (17) were conducted face-to-face in private consultation rooms at the ACCHSs, with two interviews conducted over the phone. Patients with diabetes from each ACCHS were invited by an AHW to participate in a focus group. A total of 16 patients from DAHS, ACCHS-3 and ACCHS-4 participated in the focus groups. A focus group at ACCHS-2 was cancelled due to a funeral and could not be rescheduled due to time constraints.

Digital recordings and notes from interviews and focus group sessions were reviewed and ideas and quotes transferred to Word 2007 (Microsoft, Seattle, USA). Recurring themes of potential facilitators and barriers to good quality diabetes care were identified and possible strategies for improvement in each of these identified themes were collated. Data segments from each interview and focus group were then transferred to another Word 2007 document where they were amalgamated by theme using the tabular function. Data segments were coded to be identifiable by ACCHS and profession (AHW, nurse, or GP) and to be traced back to the original document. This was an iterative process with themes and sub-themes being reviewed throughout this process. Important and recurring themes were identified, preliminary conclusions drawn and these conclusions were then tested using data from the audit.

Research notes from the “DAHS diabetes study” [28] were used to provide information on CQI processes used at DAHS during 1999–2012.

### Ethics approval

Ethics approval was granted by the Western Australia Aboriginal Health Ethics Committee (WAAHEC) and The University of Western Australia Human Research Ethics Committee. Consent was given by the health committee of each participating community.

## Results

### Audit findings

DAHS, ACCHS-2 and ACCHS-3 had high service delivery rates for most diabetes care processes, including HbA_1c_, BP and cholesterol (Table 1). ACCHS-4, however, had significantly lower rates of service provision for these care processes. Recorded service delivery rates for retinal screening, brief foot checks, full foot checks, assessment of diet and physical activity, assessment of smoking status and general practice management plans (GPMPs) were low across all health services. DAHS had better clinical outcome measurements than the other services (Table 2). Previously reported data on quality care indicators and clinical outcome measures relating to diabetes care in other Aboriginal and Torres Strait Islander populations are included in Table 1 and Table 2 [28–30, 34–37].

**Table 1:** **Proportion of patients for whom diabetes management activities were recorded in the electronic patient and information recall system during the audit period, and corresponding values from the final years of other studies**

	Study sites					National indigenous PHC ^§^	DAHS 2008-09 ^¶^
	DAHS	ACCHS-2	ACCHS-3	ACCHS-4	All ACCHS		
No. of patients included in the study	156	76	87	29	348		
Proportion female	66%	79%	57%	55%	66%		
Median age	55	52	46	55	52		
Age range	22 - 88	25 - 88	19 - 82	30 - 87	19 - 88		
Blood pressure^†^	80%	80%	84%	55%*	79%	70-88	80%
HbA_1c_^†^	71%	82%	59%	28%	66%	56-74	74%
Weight^†^	72%	70%	56%*	48%*	66%	51-77	74%
Urine protein^‡^	53%	49%	52%	28%*	50%	42-63	
ACR^‡^	67%	72%	71%	31%*	66%	46-65	81%
eGFR^‡^	94%	89%	87%	48%*	88%		94%
Cholesterol^‡^	79%	76%	71%	38%*	73%		91%
Liver function tests^‡^	92%	87%	83%*	48%*	85%		
Full foot check^‡^	14%	5%*	5%*	3%	9%	4-40	
Retinal screen^‡^	35%	26%	24%	0%*	28%	29-56	
Diet and physical activity^†^	33%	22%*	1%*	0%	20%	19-56	
Smoking assessment^†^	48%	28%*	1%*	7%*	28%	24-92	85%
GPMP^‡^	27%	11%*	15%*	28%	20%		

DAHS = Derby Aboriginal Health Service. PHC = primary health care. HbA_1c_ = glycated haemoglobin. eGFR = estimated glomerular filtration rate. ACR = albumin creatinine ratio. GPMP = general practice management plans.

*Significant difference compared with DAHS p < 0.05.

^§^Australian Aboriginal and Torres Strait Islander patients treated in PHC settings [30, 34–36].

^**¶**^Final year of the “DAHS diabetes study” [28].

^†^Recorded from 1 Jan 2012 to 30 June 2012.

^‡^Recorded from 1July 2011 to 30 June 2012.

**Table 2:** **Clinical outcome measures and proportion of patients whose outcome values met recommended clinical targets, and corresponding values from the final years of other studies**

	Study sites					National indigenous PHC ^‡^	DAHS 2008-09 ^¶^
	DAHS	ACCHS-2	ACCHS-3	ACCHS-4	All ACCHSs		
Median annual clinical outcome measures							
HbA_1c_ (%)	8	8.5	8.7^†^	8.1	8.2	8.8-9.2	8.0
BP systolic (mmHg)	127	130	125	135*	128	126-133	120
BP diastolic (mmHg)	77	80	80^†^	83*	78	77-80	75
Total cholesterol (mmol/L)	4.4	4.6	4.8*	4.2	4.5	4.7-4.8	4.5
ACR	8.7	15.6	44.1*	3.6	15.6	5.9-7.1	
Proportion of study population with median values that met targets							
HbA_1c_ ≤7%	31%	25%	16%*	17%	26%	16-32	34%
BP ≤130/80**	53%	47%	48%	19%*	38%	28-64	69%
Total cholesterol ≤4 mmol/L	39%	42%	19%*	27%	34%	29	25%
ACR <3.6 mg/mmol	38%	29%	16%*	44%	30%	38	

DAHS = Derby Aboriginal Health Service. PHC = primary health care. HbA_1c_ = glycated haemoglobin. BP = blood pressure. ACR = albumin creatinine ratio.

^‡^Australian Aboriginal and Torres Strait Islander patients treated in PHC settings [29, 30, 34–37].

^**¶**^Final year of the “DAHS diabetes study” [28].

*Statistically significant difference (P ≤ 0.05) when compared with ACCHS 1.

^†^Approached statistical significance when compared with DAHS (P = 0.05).

^**^At least half of each participant’s recorded measurements met the recommended target for both systolic and diastolic BP.

### Validity of audit findings

At each location the validity of the audit results was discussed with clinical staff. It was identified that some activities needed to be manually entered and these were not always being recorded on MMEx once completed (eg. retinal screening, smoking status assessments, diet and physical activity assessments, and foot checks) and it was believed that the data for these care processes was a long way from complete. Changes to both the way the clinic used the system and to the system itself have been implemented since the study to improve data capture and performance in these areas. Pathology results are automatically transferred to MMEx and BP if entered correctly into the progress notes is automatically captured and these data are more likely to be complete. The interviews and focus groups of clinic staff and patients outlined some suggestions for improvement of diabetes care which included:

1. The development of guidelines on how to use MMEx to recall patients for diabetes reviews, and staff education on how to do this,
2. Clarification of roles for organising recalls and undertaking particular investigations and assessments such as regular team meetings to facilitate and maintain these roles,
3. Increase staffing to allow a greater capacity for chronic disease management. Particularly more GP availability for chronic disease reviews,
4. Support for AHWs to increase the capacity of their role in diabetes management. Specifically: training in self-management support approaches; use of retinal camera and point-of-care HbA_1c_ analyser; and community engagement initiatives,
5. Continue with ongoing improvements to MMEx to increase ease-of-use to improve data capture and auditing capabilities, and to better track patients as they move between communities in the region to ensure on-going care, and
6. Engagement with allied health providers to determine approaches to improving coordination of care between services. This may include integration of assessment of the need for allied health review during a diabetes consultation, improving communication of this referral to the allied health practitioner, and notifying the patient of the allied health review appointment.

A summary of the quantitative results is shown in Table 3.

**Table 3:** **Comparison of quantitative and qualitative results**

	DAHS	ACCHS-2	ACCHS-3	ACCHS-4
**Service delivery rates** ^*^	++	++	++	-
**Clinical outcome measures** ^**†**^	++	+	-	N/A
**Recall system** ^**‡**^	++	+	-	-
**Allocated CD clinic** ^**‡**^	++	+	-	-
**AHW involvement** ^**‡**^	++	+	+	+
**Allied health integration** ^**‡**^	-	+	-	-

*++ = High, + = Moderate, − = Low.

^†^++ = Good, + = Moderate, − = Poor.

^‡^++ = Done well, + = Done to some extent, − = Not done.

N/A = not applicable as clinical outcome measures at ACCHS-4 unable to be interpreted due to very small patient numbers.

### Efficient diabetes recall systems

Clinical staff reported using different systems to recall patients. DAHS had a well-established paper-based recall system managed by an allocated RN and AHW that identified all known regular patients with diabetes. MMEx was searched intermittently to identify any patients missed in the paper system. If patients had not had a diabetes review in more than three months they were notified by letter and a flexible appointment was made. Transport to the clinic was offered to the patients if required.

ACCHS-2 had started a recall system for diabetes in the early months of the audit period, and these efforts increased over the course of the audit. This program was run by an RN who was able to dedicate time most days to this process. Patients who were more than three months overdue for HbA_1c_ testing were identified on MMEx, notified in person by an Aboriginal outreach worker, and asked to present to the clinic that day or as soon as convenient. Transport was offered if required.

At both ACCHS-3 and −4 there was no formal recall system for diabetes reviews, but some GPs performed intermittent searches of MMEx to identify patients who were due for recall and then asked the AHW and/or RN to notify these patients and initiate a diabetes review. This largely doctor based system reportedly worked more effectively at ACCHS-3 due to more consistent medical staff compared with ACCHS-4 which only had two visits by doctors per week and less follow through by staff on the ground.

Across all ACCHSs factors that were identified as real or potential facilitators to effective diabetes recall included the provision of patient transport and the involvement of an AHW or Aboriginal outreach worker in the recall process. High staff turnover, a lack of clarity over whose role it was to manage the recall system, and uncertainty over how to use MMEx to extract a recall list were identified as barriers to a functioning diabetes recall system at ACCHS-3 and −4. Staff reported that functioning recall systems at DAHS and ACCHS-2 contributed to the high service activity at these services. Staff at ACCHS-4 reported the lack of an effective recall system was the main factor contributing to the low service activity (Table 1).

### Allocated chronic disease roles for diabetes review

At DAHS and ACCHS-2 there were allocated chronic disease ‘clinics’ where diabetes reviews took place. At DAHS this was a long standing arrangement and involved an RN, AHW and GP who saw patients specifically for chronic disease reviews two days per week. On the other days the RN and the AHW would organise recalls and undertake home visits to selected patients.

At ACCHS-2 this was a new system and involved an RN who saw patients for chronic disease reviews five days per week. Patients were sometimes referred on to see the GP and this meant returning to the waiting room or returning on another day.

Having a separate chronic disease clinic resulted in reduced waiting times for patients being seen for diabetes reviews and was looked upon favourably by staff and patients from both of these ACCHSs. Both staff and patients at DAHS pointed out that the community was well aware of the chronic disease clinic and the importance of attending for regular review. Staff at ACCHS-2 reported that patients were becoming more aware of the chronic disease clinic run by the RN and that this was increasing attendance rates for diabetes review. There were also regular team meetings at DAHS and ACCHS-2 where staff were able to discuss any issues regarding chronic disease management and it was believed that this contributed to well-coordinated diabetes care.

ACCHS-3 and ACCHS-4 had allocated GP time for chronic disease management. Staff from these ACCHSs explained that acute presentations intruded on chronic disease time for their GPs and that lack of coordination of chronic disease management reduced the effectiveness of these programs.

Barriers to well-coordinated diabetes care identified at ACCHS-3 and ACCHS-4 included high nursing staff turnover; a lack of clarity over roles in regards to who should undertake the various diabetes service activities; and a lack of regular team meetings. GPs suggested that in order to improve the coordination and efficiency of diabetes care at these ACCHS effective patient recall followed by initial review with an AHW and/or RN for certain diabetes service activities and self-management support was needed prior to GP review.

### Support and involvement of Aboriginal health workers in diabetes care

At all health services, GPs, RNs, AHWs and patients identified the involvement of AHWs as an important facilitator to delivering good diabetes care. AHWs were identified as important in breaking down the communication and cultural barriers between Aboriginal patients and non-Aboriginal health care staff and helped to make patients feel more comfortable in the clinic. Patients from DAHS appreciated the involvement of the AHWs in the chronic disease clinic and the staff believed that this close teamwork helped them to learn skills from one another.

At the other three services, there was limited involvement of AHWs in diabetes care. AHWs often took patient observations prior to the patient being seen by the RN or GP and were rarely involved in these consultations. Staff from these services stated that an increase in AHW involvement in diabetes care, particularly in self-management support around healthy lifestyle and smoking cessation, as well as their involvement in the consultations would be beneficial for both patients and staff. AHWs could learn clinical skills from the GPs and RNs and non-Aboriginal staff members could learn more culturally appropriate ways of tackling a diabetes consultation from the AHWs. Staff stated that for this to happen AHWs would need to be provided with more training as well as on-site support from RNs and GPs.

At ACCHS-3 a retinal screening program run by an AHW was identified as a great success (although not all of this data had been recorded in MMEx and therefore is not accurately represented in this study). Staff at this service believed that this role could be extended with the development of an allocated room designated as an AHW ‘space’ for undertaking diabetes reviews. Unfortunately at ACCHS-2, −3 and −4 clinical space was overcrowded and lack of physical space meant both RNs and AHWs did not always have a room. Staff reported that care often took place in curtained off bays and patients reported that this reduced privacy and was a deterrent to diabetes care.

### Well-coordinated integration with allied health professionals

Various allied health professionals including diabetes educators, dieticians, podiatrists and optometrists visited each of the ACCHSs at variable time intervals. Seven of 17 staff interviewed as well as patients from each focus group identified allied health professionals as important in delivering diabetes care, particularly diabetes educators and dieticians.

At three of the four ACCHSs allied health referral management was reportedly not functioning well. Allied health staff all worked for different organisations and their visits to the ACCHSs were not always well coordinated. Problems included referrals not being made, referrals not being received by the allied health professional, patients not being notified of allied health visits, and patients not being available when allied health staff visited. In addition some allied health workers relied on their own recall lists, providing good care to selected patients but not working well with the clinic to identify all eligible patients.

Staff from ACCHS-2 on the other hand described an allied-health referral system which functioned very well. An Aboriginal administration staff member was notified of the need for a referral and liaised with the allied health professional, managed the appointment system and organised recall letters to be delivered to the patients well in advance of their appointment. Staff explained that there were still issues with not all patients being offered allied health referral, however this was improving with involvement of the chronic disease RN.

### The process of conducting CQI in the Kimberley

DAHS has a long history of conducting CQI [28]. During the “DAHS diabetes study” staff reported concern over the size of the “off the shelf” audit tools that were briefly used at DAHS and their labour intensiveness such as Australian Best Practice for Chronic Disease project (ABCD, now the one21seventy project) and the Australian Primary Care Collaboratives program (APCC) [31, 38]. They also highlighted the need to 1) incorporate additional qualitative elements which better capture features of Kimberley service provision; 2) embed health services monitoring and audit practices into routine health service operation, rather than their application as extra tasks carried out intermittently; and 3) for a system of evaluation that is flexible and responsive to variations between services, and within services over time.

Three staff members interviewed from ACCHS-4 suggested that regular internal reporting by ACCHSs to KAMSC would help improve their diabetes care by ensuring ACCHSs are more accountable for their performance in regards to chronic disease care.

### Recommendations

Recommendations from this study to help improve the quality of care provided by Kimberley ACCHS include: 1) system changes to MMEx to increase the rates of data capture, increase tracking of patients as they move between communities in the region, and improve ease-of-use; 2) allocation of staffing resources where needed and clearer description of roles and responsibilities with particular regard to recall systems and chronic disease management programs; and 3) development of a role for a KAMSC regional ‘CQI facilitator’ to assist ACCHS with their CQI efforts. Features of effective quality improvement strategies for chronic disease management in Aboriginal Community Controlled Health Services as identified in this study include:

1. Health service ownership of the quality improvement cycle. Integration of the cycle into standard practice,
2. An audit tool that can be feasibly adapted to address the perceived needs of the health service,
3. Whole-of-service involvement in interpretation of audit results,
4. Involvement with staff and community in developing strategies for improvement,
5. Support from regional organization to implement strategies for change.

## Discussion

This study deepens our understanding of what is required to develop a sustainable and practical approach to CQI in Aboriginal health services. It extends and adapts the successful experience of one ACCHS over 12 years to other remote environments, documents differences in results and relates these to systems at a local level. This study documented core features that contributed to well-coordinated diabetes care: allocated roles for chronic disease management; a well-functioning recall system; the involvement and support of AHWs in diabetes care delivery; and well-coordinated integration with allied health professionals. Difficulties in these and other areas have been described in the Aboriginal health care setting [22, 23, 28, 31, 36, 39–46]; a situation that is complex with no ‘one size fits all’ solutions. Despite these difficulties, ACCHSs are equipped to tackle such issues through sophisticated quality improvement and chronic disease management programs such as we describe in this paper.

Diabetes care was better overall at DAHS as shown by higher service delivery rates and generally better clinical outcome measures. Two other ACCHSs were reasonably similar to DAHS with diabetes care service delivery rates, but had worse outcomes in terms of BP, HbA_1c_ and cholesterol. These differences may relate to more recent or intermittent systems at these two ACCHS and/or to other differences between the communities concerned. The fourth ACCHS had substantially less complete follow up. Clarity of roles and responsibilities for diabetes management, combined with effectively structured diabetes care delivery systems contributed to good diabetes care in this study. Effective recall systems with buy in from staff led to high service delivery rates, and have been shown to be fundamental to chronic disease management for both the Aboriginal and Torres Strait Islander and the wider health sectors [23, 39, 47]. DAHS had a high level of involvement and support of AHWs in diabetes management which contributed to better levels of care, as has happened elsewhere [22, 23, 40]. There are many barriers to AHW involvement in chronic disease care that have been previously described including issues surrounding training, role definitions, and relationships with non-Indigenous health care staff [39–41, 48]. In order to attempt to overcome these barriers and improve AHW involvement in diabetes care in the other ACCHSs, considerable ongoing support from management will be required. Allied health worker involvement can contribute to better diabetes care, but a need for better integration and coordination of allied health worker involvement was identified, this has also been expressed in the literature in the Aboriginal health sector [43]. Sustaining improvements over time in each of these areas using CQI is however the greatest challenge [49].

We found that the main features of effective and sustainable CQI include seamless and timely data collection as well as local ownership of the process based around openness to admitting deficiencies and embracing change. We found that in order to facilitate change this requires more than an external report on processes and indicators (as with ABCD/one21seventy or the APCC) [31, 38], but rather on the ground support for health services to implement and improve their own CQI. We explored the local knowledge about what was and was not working at the health service level, and what was required to improve it, rather than using the time laborious Systems Assessment Tool processes of ABCD/one21seventy [50].

It has been suggested that in order to support health service delivery planning and action for improvement at the local and regional level, careful interpretation of the factors underlying performance variation is required [30]. Local ownership of the CQI process and a trust in local knowledge about what works, and what is needed to make it work better is crucial for this to occur.

In the model we used, the steps were: support an individual service to make progress with their own CQI; share the experience with a small number of other health services with connections or similarities; and continue to review the CQI process and share these experiences to help develop regional strategies for CQI that are flexible and adaptable. This process is ongoing.

Based on the success of CQI at DAHS, which has been going for over 12 years, we argue that a locally owned CQI process is more sustainable than an externally driven program as it is not reliant on external organisations for expertise and tools that are not freely shared and adaptable, and which build expertise within organisations other than those responsible for ongoing service delivery. Locally owned CQI can be tailored to meet the individual needs of a health service and health service staff are more likely to be engaged with the process, leading to improved CQI effectiveness and sustainability. This may involve adapting existing CQI tools to suit local needs.

This could be overseen by a ‘CQI facilitator’ who would support health service staff to undertake CQI activities. This use of CQI facilitators is supported in the literature [45], however we argue that this role would work more effectively if based at a regional or state ACCHS affiliate level, and needs to be someone who is trusted and engages with the health services and staff on a long-term basis. If government funding could focus on developing and supporting these roles, we argue that this would be a more cost-effective approach to improving CQI that would result in a greater improvement in outcomes than is currently the case.

A limitation of this study is that it only describes one cycle of CQI and therefore does not describe changes over time; however this has already been done at DAHS [28]. A longer term interventional study is needed in order to continue to review and refine the CQI approach taken in this study, and to evaluate changes in service delivery rates and clinical outcome measures.

## Conclusion

The expansion of a long-term locally driven CQI process from one service to three others in the Kimberley region has identified the core features that contribute to well-coordinated diabetes care (dedicated staff for chronic disease management; the involvement and support of AHWs in diabetes care delivery; a well-functioning recall system; and well-coordinated integration with allied health professionals). The process described in this paper provides practical examples that local services can adopt to improve their chronic disease care. Locally run CQI processes are likely to be more responsive to individual needs of health services by providing health service staff and community members with a greater sense of ownership of the process; it also ensures that CQI is independently maintained into the future through local networks.

## Abbreviations

ABCD: Audit and Best Practice for Chronic Disease
ACCHS: Aboriginal Community Controlled Health Service
ACR: Albumin creatinine ratio
AHW: Aboriginal Health Worker
APCC: Australian Primary Care Collaboratives
BP: Blood pressure
CQI: Continuous quality improvement
DAHS: Derby Aboriginal Health Service
eGFR: Estimated glomerular filtration rate
GP: General practitioner
GPMP: General practice management plan
HbA_1c_: Glycated haemoglobin
KAMSC: Kimberley Aboriginal Medical Services Council
LFT: Liver function test
T2DM: Type 2 diabetes mellitus
RN: Registered nurse.
